# Post-stroke inflammation—target or tool for therapy?

**DOI:** 10.1007/s00401-018-1930-z

**Published:** 2018-11-27

**Authors:** Kate Lykke Lambertsen, Bente Finsen, Bettina Hjelm Clausen

**Affiliations:** 1 Department of Neurobiology Research, Institute of Molecular Medicine, University of Southern Denmark, 5000 Odense, Denmark; 2 Department of Clinical Research, BRIDGE-Brain Research-Inter-Disciplinary Guided Excellence, University of Southern Denmark, 5000 Odense C, Denmark; 3 Department of Neurology, Odense University Hospital, 5000 Odense, Denmark

**Keywords:** Cytokines, Ischemia, Immune therapy, Drugs, Neuroprotection

## Abstract

Inflammation is currently considered a prime target for the development of new stroke therapies. In the acute phase of ischemic stroke, microglia are activated and then circulating immune cells invade the peri-infarct and infarct core. Resident and infiltrating cells together orchestrate the post-stroke inflammatory response, communicating with each other and the ischemic neurons, through soluble and membrane-bound signaling molecules, including cytokines. Inflammation can be both detrimental and beneficial at particular stages after a stroke. While it can contribute to expansion of the infarct, it is also responsible for infarct resolution, and influences remodeling and repair. Several pre-clinical and clinical proof-of-concept studies have suggested the effectiveness of pharmacological interventions that target inflammation post-stroke. Experimental evidence shows that targeting certain inflammatory cytokines, such as tumor necrosis factor, interleukin (IL)-1, IL-6, and IL-10, holds promise. However, as these cytokines possess non-redundant protective and immunoregulatory functions, their neutralization or augmentation carries a risk of unwanted side effects, and clinical translation is, therefore, challenging. This review summarizes the cell biology of the post-stroke inflammatory response and discusses pharmacological interventions targeting inflammation in the acute phase after a stroke that may be used alone or in combination with recanalization therapies. Development of next-generation immune therapies should ideally aim at selectively neutralizing pathogenic immune signaling, enhancing tissue preservation, promoting neurological recovery and leaving normal function intact.

## Ischemic stroke

Ischemic stroke is the second leading cause of preventable deaths and the third leading cause of long-term disability worldwide [84]. This review focuses on the possibility of targeting post-stroke inflammation to improve tissue preservation, neurological outcome, and long-term survival. Ischemic stroke, accounting for approx. 90% of all stroke cases [84], is caused by embolism or thrombosis of a cerebral artery. This typically occurs in the middle cerebral artery (MCA), which supplies the lateral convexity of the cerebral hemisphere and thereby the majority of the primary motor and sensory cortex, leading to contralateral hemiplegia with reduced sensation. Today, recanalization by intravenous (i.v.) thrombolysis and thrombectomy are first-line treatments for ischemic stroke patients [95]. One of the major criteria for i.v. thrombolysis is the 4.5-h ‘therapeutic time window’, although the recent DAWN and DEFUSE 3 trials, which combine thrombectomy and i.v. thrombolysis, suggest expanding the therapeutic window up to 24 h when using perfusion imaging to guide treatment [2, 127]. Importantly, these studies additionally document that restoring perfusion not only leads to smaller infarcts, but that smaller infarcts correlate with a better neurological outcome [2, 127]. Given the low number of stroke patients eligible for treatment using thrombolysis and/or thrombectomy (approx. 10%), novel treatment options are critically needed. New therapies targeting key pathogenic mechanisms, including post-stroke inflammation, are currently being pursued experimentally and clinically, either alone or in combination with thrombolysis and/or thrombectomy [23]. Such treatments might also benefit stroke patients with good collateral blood supply who suffer permanent ischemia or patients in whom recanalization treatment is contraindicated.

## The ischemic penumbra as target for post-stroke intervention

The ischemic penumbra consists of electrophysiologically silenced, potentially salvageable tissue [7], that can be assessed clinically using the “mismatch” between perfusion- and diffusion-weighted magnetic resonance images (PWI–DWI mismatch) [142] or positron emission tomography (PET) [77]. The cerebral metabolic rates for oxygen measured by PET define cerebral blood flow in cortical grey matter below 12 ml/100 g/min as infarct core, flow between 12 and 22 ml/10 g/min as critically hypoperfused penumbral tissue, and flow between 22 and 35 ml/100 g/min as an area of oligemia, i.e. hypoperfused tissue without risk for infarction [77]. This should be compared to the flow in normal grey matter, which is between 50 and 55 ml/100 g/min [95]. In tissue sections, the penumbra is defined as areas with reduced protein synthesis but preserved ATP content. This matches brain areas with transient heat shock protein 70 mRNA expression from 3 to 4 h after MCA occlusion (MCAO) [74, 75]. Using these definitions, the penumbra presents 30% of the final infarct volume at 1 h, approx. 18% at 6 h, and 5% at 24 h after permanent MCAO (pMCAO) [74]. After transient MCAO (tMCAO), the penumbra is initially increased as a result of edema associated with reperfusion, after which it is gradually recruited into the infarct and regresses to the final infarct volume at day 3 [75]. In rats, the infarct volume measured at 24 h after proximal pMCAO is significantly larger than after 60 min of proximal tMCAO, but is similar to that observed after 180 min of proximal tMCAO [112].

By showing that the therapeutic window can be expanded, the DAWN and DEFUSE 3 trial results, combining the use of thrombectomy and thrombolysis [2, 127], have ‘thrown the ball back in the ring’ in experimental stroke research. Some tMCAO models mirror thrombectomy in terms of reperfusion dynamics (review by [107]), encouraging testing of novel combination treatments. Furthermore, the clinical documentation that smaller infarcts translate into better neurological outcome [2, 127] emphasizes the importance of infarct volume reduction, ideally in conjunction with improved functional recovery, as an important outcome in experimental stroke research. The size of ischemic damage is typically presented as: 1—total infarct volume (‘direct infarct volume’ given in mm^3^), or 2—percentage of infarcted tissue in the ipsilateral hemisphere, corrected for edema formation and infarct resorption (‘indirect infarct volume’) (for details see [140]). Infarct volumes given as percentages and corrected for edema/resorption remain largely constant from 24 h to 24 weeks [140]. Direct infarct volumetric data obtained at 24 h after occlusion are robust, while data obtained at 5 days represents the cumulative effect of infarct formation and resorption [94, 140].

## The inflammatory response in stroke

**Fig. 1:**
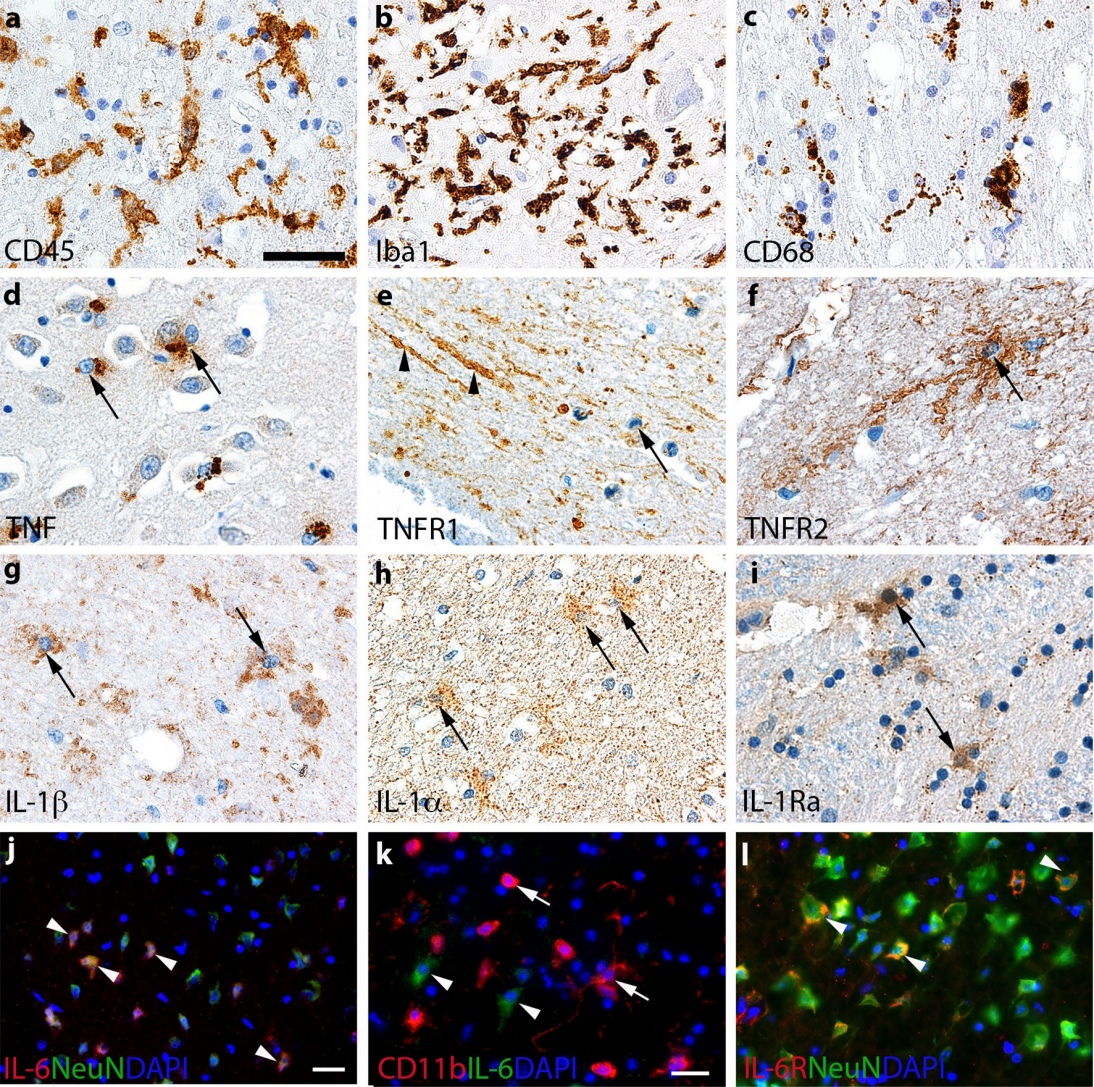
Neuroinflammation in the post-ischemic human and murine brain. **a**–**c** Immunohistochemical staining of CD45^+^ (**a**), Iba1^+^ (**b**), and CD68^+^ (**c**) microglia/macrophages in human post-mortem ischemic brain tissue. **d**–**i** Immunohistochemical staining of TNF^+^ (**d**), TNFR1^+^ (**e**), TNFR2^+^ (**f**), IL-1β^+^ (**g**), IL-1α^+^ (**h**), and IL-1Ra^+^ (**i**) cells in human post-mortem ischemic brain tissue. (**j, k**) Immunofluorescence double staining showing co-localization of IL-6 to NeuN^+^ neurons (**j**), but absence of IL-6 to CD11b^+^ microglia/macrophages (**k**) in the murine brain after pMCAO. **l** Immunofluorescence double staining showing co-localization of IL-6R to NeuN^+^ neurons in the murine brain after pMCAO. Unpublished images of CD45, Iba1, CD68, TNF, TNFR1, TNFR2, and IL-1Ra stained tissue sections were acquired from human post-mortem ischemic brain tissue processed as previously described [31, 33] using already published protocols, except for IL-1β and IL-1α. Staining for IL-1β and IL-1α was performed using similar protocols and the following antibodies: Human IL-1α Ab (monoclonal mouse IgG_2A_, clone #4414, 1:1,200, R&D Systems) and human IL-1β Ab (monoclonal mouse IgG1, clone #2E8, 1:50, BioRad). Unpublished images of IL-6 and IL-6R co-localized cells were acquired from parallel tissue sections from mice subjected to pMCAO as described in [70]. In images **a**–**i**, Toluidine blue was used as a counterstain and in **j**–**l**, DAPI was used as a nuclear marker. Scale bars: **a**, **i** = 40 μm, **j** = 20 μm, and **k**, **l** = 20 μm. *IL* interleukin, *IL-6R* interleukin-6 receptor, *TNF* tumor necrosis factor, *TNFR* tumor necrosis factor receptor. The use of human brains was approved by the Danish Biomedical Research Ethical committee for the Region of Southern Denmark (permission number S-20080042) as stated in the original references

Inflammation is integral to the pathophysiology of ischemic stroke and a prime target for the development of new stroke therapies. The first immune cells to sense a stroke are the brain-resident microglial cells, which are innate immune cells that are perfectly situated and equipped to sense imbalances in the CNS. Microglia express receptors that are involved in immune signaling and modulation, recognition of danger signals elicited from dying cells, pathogens and self-antigens, as well as neurotransmitter receptors in both human [56] and mouse [78]. Like other cells, the microglia are sensitive to ischemia. 12 h after pMCAO, CD11b^+^ microglia in the infarct show signs of fragmentation, and by 24 h the number of microglia within the infarct is reduced [81, 94]. Microglia in the ‘peri-infarct’ show signs of activation in the form of process retraction from 30 min to 1 h after pMCAO, followed by upregulation of CD11b, CD45 and Iba1 in the peri-infarct from 3.5 to 6 h [32, 81, 94], where also the first CD11b^+^ macrophage-like cells (and Gr1^+^ neutrophils) appear [32, 94]. Microglial activation in the peri-infarct persists weeks after MCAO [94, 131]. Importantly, the microglia in the peri-infarct and infarct display different pro- and anti-inflammatory phenotypes [32, 33, 115], which include the expression of the pro-inflammatory cytokines tumor necrosis factor (TNF), interleukin (IL)-1β, and the anti-inflammatory IL-1 receptor antagonist (IL-1Ra) (Fig. 1) [32, 33, 92]. Microglia appear not to display classical M1 and M2 phenotypes after experimental stroke [61]. During later stages microglia, like monocytic macrophages, contribute to the resolution of the infarct by phagocytosing dead cells or debris, which is considered beneficial (review by [124]). However, microglia can also engulf viable ischemic neurons, that transiently express “eat-me” signals [122], and if dysregulated thereby increase neuronal cell death in the peri-infarct.

The infiltrating leukocytes, predominantly polymorphonuclear leukocytes (PMNs, neutrophils) and monocytes/macrophages, play different and complex roles in ischemic stroke. Neutrophils infiltrate early after MCAO [26]. They attach to the endothelium by binding different adhesion molecules (review by [125]), and with CXCL1 and CXCL2 as the main chemokines responsible for neutrophil extravasation [176]. Neutrophils expressing Ly6G and myeloperoxidase have been identified in the leptomeninges from 6 h after occlusion, thereafter in the Virchow–Robin spaces and superficial cortical layers, to eventually become widespread in the infarct and peri-infarct [133, 176]. In rodent pMCAO models, the number of neutrophils in infarct and peri-infarct peaks at 24 h and gradually decreases from 48 to 72 h [133, 176]. Differences in the peak of neutrophil recruitment have been reported between pMCAO and tMCAO [198]. Neutrophil accumulation has traditionally been considered detrimental post-stroke, either through the release of neurotoxic proteolytic enzymes [4] or neutrophil accumulation causing further blood flow obstruction and the ‘no-reflow’ phenomenon (reviewed in [39]). Neutrophils have also been shown to cause disruption of the blood–brain barrier (BBB) and hemorrhagic transformation post-stroke, worsening the neurological outcome [83]. Blockade of neutrophil recruitment has been shown to improve the functional outcome in rodent stroke models [83]. Neutropenia does not affect infarct size after MCAO [76] however, and none of the anti-neutrophil therapies tested so far have shown a beneficial effect in stroke patients [83]. Interestingly, neutrophils appear to display different phenotypes (neurotoxic N1 and neuroprotective N2) that may shape the effector functions of other cells and they are themselves cleared by phagocytic microglia/macrophages, which is considered important for the resolution of inflammation post-stroke [36]. Therefore, inhibiting neutrophil recruitment could also prove damaging if applied at the wrong time point.

Recruitment of circulating monocytes to the ischemic brain equals that of neutrophils and is regulated by adhesion molecules, chemokines, and cytokines. CD11b^+^Ly6C^high^CCR2^+^ monocytes appear to be the predominant cell type recruited after both pMCAO and tMCAO [27, 116]. Recruitment after tMCAO takes place in a CCR2-dependent manner [41], while this appears not to be the case after pMCAO [27]. Histologically, CD11b^+^ and CD45^+^ macrophage-like cells are observed both in the infarct and peri-infarct from 6 to 48 h after pMCAO [94, 131]. From 3 to 7 days after occlusion the infarct becomes infiltrated with CD11b^+^, CD45^+^, and ED1^+^ macrophages, reminiscent of phagocytic ‘foam cells’ that are prominent in the infarct [81, 94]. Interestingly, when in the brain the Ly6C^high^CCR2^+^ monocytes change their phenotype by downregulating Ly6C expression, upregulating F4/80, and then expressing arginase-1 and the chitinase-like protein YM-1, thereby developing into M2 phenotype macrophages [116]. This occurs from 1 to 3 days after pMCAO [116]. Histologically, Ym1^+^ and CD206^+^ cells have been shown to be abundant within the infarct core at 24 h, and to be even more numerous at 7 days, along with cells expressing the lysosomal marker CD68 [131]. This is in line with a role in infarct resolution and repair.

Although monocytes/macrophages have been reported to exacerbate ischemic brain damage in the acute phase after tMCAO [41], blocking the infiltration of Ly6C^high^ monocytes (and neutrophils) using a CCR2 antagonist worsened the outcome after tMCAO, which was ascribed to CCR2 antagonism altering the polarization of infiltrated macrophages [27]. Monocytes/macrophages have been suggested to exert beneficial effects in the sub-acute phase after a stroke, by preventing hemorrhagic transformation [63], emphasizing that inhibition of monocyte recruitment might be damaging if done at the wrong time point. To add to the complexity, it appears that subsets of CD11b^+^CD45^high^ macrophages express different pro- and anti-inflammatory cytokines at different time points after pMCAO [27, 32, 33, 92], raising the potential to modulate this expression and to stimulate the production of anti-inflammatory cytokines such as IL-1Ra [33]. The emerging understanding of how macrophages are stimulated by the ischemic environment to adopt distinct phenotypes or exert different functions might reveal new therapeutic strategies for controlling inflammation after ischemic injury.

Recent studies have also implicated lymphocytes in the pathogenesis of acute stroke. Since it is largely unknown as yet how these cells affect inflammation in the ischemic brain, the reader is referred to existing reviews on this topic [153].

## Cytokines and cytokine therapies in experimental and human stroke

Treatment strategies aimed at preventing ischemia-induced cell death and promoting anti-inflammatory responses in ischemic tissue at risk have been studied both experimentally and in clinical trials (Table 1). Special attention has been given to inflammatory cytokines and the possibility to modulate their pro- or anti-inflammatory properties. Cytokine therapies are based on administration of highly specific engineered antibodies, soluble cytokine receptors, and mutant or fusion proteins that bind and neutralize the activities of a given cytokine (Table 2). A number of drugs targeting the key pro-inflammatory cytokines TNF, IL-1, and IL-6 (Table 2) are already being used in patients for the treatment of non-neurological diseases such as rheumatoid arthritis, inflammatory bowel disease, and psoriasis. As cytokines have both beneficial and detrimental effects, their neutralization can result in unwanted side effects, including predisposing patients to infections, lupus-like syndrome, lymphoma, long-term effects on the cardiovascular system, and demyelinating disease [151]. Therefore, there is a need to develop and evaluate novel therapeutics that can better distinguish between detrimental and protective signaling of a given cytokine. Four cytokines have proven especially promising as potential therapeutic targets in experimental ischemic stroke: the pro-inflammatory cytokines TNF, IL-1, IL-6 and the anti-inflammatory cytokine IL-10.

**Table 1:** Studies on anti-cytokine treatments in experimental and human stroke

Ischemia model	Strain	Intervention	Results	Target involved	References
TNF system					
Mouse					
Distal pMCAO (electrocoagulation)	C57BL/6	i.v. injection of 10 mg/kg anti-TNF inhibitor (etanercept) or 10 mg/kg anti-solTNF inhibitor (XPro1595) 30 min after occlusion	No change in infarct volume, improved functional outcome	tmTNF and/or solTNF	[30]
Proximal tMCAO (60 min, filament)	C57BL/6	i.v. injection of 1 mg/kg etanercept or cTfRMab-TNFR 45 or 90 min after occlusion	cTfRMAb-TNFR decreased infarct volume and neural deficits	tmTNF and solTNF	[167]
Proximal tMCAO (60 min, filament)	C57BL/6	i.v. injection of 1 mg/kg cTfRMab-TNFR and 1 mg/kg cTfRMab-GDNF 45 min after occlusion	cTfRMAb-TNFR and cTfRMAb-GDNF decreased infarct volume	mTNF and solTNF	[168]
Cortical photothrombosis (i.v. Bengal Rose injection followed by 20 min focal illumination)	C57BL/6	Intracortical infusion of 1 μg/day sTNF-α- R1 for 1 week	sTNF-α- R1 preserved post-stroke deprivation-induced brain plasticity	solTNF (and tmTNF)	[98]
Distal pMCAO (electrocoagulation)	BALB/c	i.p. or i.v. injection of 3 mg/kg TNF-bp immediately after occlusion	TNF-bp decreased infarct volume	tmTNF and solTNF	[120]
Distal pMCAO (electrocoagulation)	BALB/c	Topic administration of 3 mg/kg TNF-bp immediately and 1 h after occlusion	TNF-bp decreased infarct volume	tmTNF and solTNF	[121]
Rat					
Proximal tMCAO (90 min, filament)	Wistar	i.p. injection of 7 mg/kg chimeric anti-TNF mAb (infliximab) or 5 mg/kg anti-TNF (etanercept) 0 and 6 hrs after occlusion	Infliximab and etanercept decreased infarct volume	tmTNF and solTNF	[5]
Proximal tMCAO (120 min, filament)	SD (diabetic and non-diabetic)	i.p. or i.v. injection of 300, 450, or 900 μg/kg anti-TNF (etanercept) within 24 hrs before or immediately after occlusion	Etanercept administered once before occlusion reduced infarct volume in non-diabetic rats and at 900 μg/kg/daily for 5 weeks prior to occlusion decreased infarct volume in diabetic rats	tmTNF and solTNF	[82]
Distal tMCAO (occluded and cut)	SHR	10 μg TNF mAb or 12.5 μg solTNFR1, 30 min before and 3 and 6 h after occlusion	TNF mAb and solTNFR1 decreased infarct volumes	tmTNF and solTNF	[8]
Proximal tMCAO (60 min, filament)	SD	i.v. injection of ex vivo-derived dendritic cells (exDCs) overexpressing solTNFR1 6 h after reperfusion	solTNFR1-exDCs decreased infarct size and inflammation	solTNF and (tmTNF)	[186]
Proximal tMCAO (120 min, filament)	SD	i.v. injection of 15 mg/kg anti-TNF mAb immediately after reperfusion	Anti-TNF mAb decreased infarct volume and edema	tmTNF and solTNF	[79]
Human					
Chronic stroke (13-36 months old)		Perispinal, interspinous, extrathecal injection of 25 mg anti-TNF (etanercept)	Neurological improvement in all patients (*n *= 3)	tmTNF and solTNF	[173]
Chronic stroke (≤3 to >120 months)		Perispinal, interspinous, extrathecal injection of 25 mg anti-TNF (etanercept)	Improved motor impairment, spasticity, sensory impairment, cognition, psychological/behavioral function, aphasia, and pain (n=617)	tmTNF and solTNF	[174]
IL-1 system					
Mouse					
Distal tMCAO (30 and 45 min, filament)	C57BL/6	s.c. injection of 100 mg/kg IL-1Ra 30 or 180 min after	IL-1Ra decreased infarct size and neurological deficit and improved functional outcome	IL-1α, IL-1β	[106]
Distal pMCAO (electrocoagulation)	BALB/c	s.c. injection of 100 mg/kg IL-1Ra 30 or 180 min after			
Distal pMCAO (electrocoagulation)	C57BL/6	i.v. injection of IL-1Ra-producing bone marrow-derived cells 30 min after occlusion	IL-1Ra-producing bone marrow-derived cells decreased infarct volumes and improved functional outcomes	IL-1α, IL-1β	[33]
Proximal tMCAO (40 min, filament)	C57BL/6	i.v. injection of IL-1Ra-producing bone marrow-derived cells 30 min after reperfusion			
Proximal tMCAO (30 min, filament)	C57xSV129	i.c.v. injection of 2.5 μg IL-1Ra or 2.5 ng IL-1β 30 min before occlusion and 10 min after reperfusion	IL-1β increased, whereas IL-1Ra decreased infarct volumes	IL-1α, IL-1β	[175]
Rats					
Proximal tMCAO (120 min, filament)	SD	i.v. injection of 50 mg/kg IL-1RA-PEP at the time of reperfusion	IL-1RA-PEP alleviated brain infarction, cerebral edema, neurological deficit score, and motor performance	IL-1β	[195]
Proximal tMCAO (filament)	SD	i.v. injection of 10 mg at the time of occlusion followed by i.v. infusion 0.8 mg/h hIL-1Ra (anakinra) for 24 hrs	Anakinra reduced infarct volume	IL-1α, IL-1β	[28]
Proximal tMCAO (120 min, filament)	Wistar	i.v. injection of 5, 10, or 20 mg/kg hIL-1Ra (anakinra) at 3, 6 or 12 hrs after after occlusion	Anakinra reduced infarct volume and improved neurological deficits dose- and time-dependently	IL-1α, IL-1β	[189]
Proximal tMCAO (120 min, filament)	SD	i.v. injection of 50 mg/kg IL-1RA-PEP at the time of reperfusion	IL-1RA-PEP alleviated brain infarction, cerebral edema, neurological deficit score and motor performance	IL-1α, IL-1β	[195]
Distal pMCAO (electrocoagulation)	SD	i.c.v. injection of 10 μg rhIL-1Ra 30 min before and 10 min after occlusion	rhIL-1Ra reduced infarct volumes	IL-1α, IL-1β	[138]
Distal tMCAO (60 min, filament)	SD	i.c.v. injection of recombinant adenovirus vector carrying the human IL-1Ra cDNA (Ad.RS*VIL-1ra*) 5 days prior to experimental stroke	Ad.RS*VIL-1ra* reduced infarct volumes	IL-1α, IL-1β	[12]
Proximal pMCAO (filament)	Wistar	i.v. injection of 100 mg/kg rhIL-1Ra immediately prior to and again s.c. 3 times per day for 7 days	rhIL-1Ra reduced infarct volumes and improved functional scores	IL-1α, IL-1β	[59]
Distal pMCAO (electrocoagulation)	SD	s.c. injection of 100 mg/kg rhIL-1Ra at 0, 4, 8, 12, and 18 h after occlusion	rhIL-1Ra reduced infarct volumes dose- and time- dependently and inhibited cerebral edema at 24 hrs	IL-1α, IL-1β	[137]
Human					
Acute stroke (< 6 h)		i.v. injection of 100 mg bolus rhIL-1Ra, followed by 2 mg/kg per hour for 72 h	rhIL-1Ra improved clinical outcomes (survival to 3 months, NIHSS, BI, and mRS scores) at 3 months (n=17)	IL-1α, IL-1β	[51]
Acute stroke (< 6 h)		i.v. injection of 100 mg bolus rhIL-1Ra, followed by 2 mg/kg per hour for 72 h	rhIL-1Ra reversed peripheral innate immune suppression in the acute phase of stroke (n=17)	IL-1α, IL-1β	[158]
Acute stroke (< 5 h)		s.c. injection of 100 mg rhIL-1Ra (anakinra) twice daily for 3 days	Anakinra reduced plasma inflammatory markers but did not affect mRS at 3 months (n=39)	IL-1α, IL-1β	[159]
IL-6 system					
Mouse					
Distal pMCAO (electrocoagulation)	C57BL/6	i.v. injection of 500 ng IL-6, solIL-6R, or 500 ng IL-6 followed by 500 ng solIL-6R 5 min or 5 and 60 min after occlusion	IL-6 injection improved behavioral outcome without affecting infarct size; co-administration of Il-6 and solIL-6R increased infarct volume, number of PMNs and impaired endurance	IL-6, IL-6R, gp130	[70]
Proximal tMCAO (60 min, filament)	C57BL/6	i.c.v. injection of 10 ng anti-IL6 mAb or intranasal administration of 0.1 μg rIL-6 every 24 h for 2 weeks starting 14 days after occlusion	Anti-IL-6 mAb reduced proliferation and neuronal differentiation of neural progenitor cells in the ipsilateral SVZ, as well as functional recovery; rIL-6 conferred the opposite effect	IL-6	[111]
Proximal tMCAO (45 min, filament)	C57BL/6	i.p. injection of 100 μg/g bodyweight IL-6Ra immediately after reperfusion	Anti-IL-6Ra increased infarct volume and affected neurological function.	IL-6R	[192]
Rats					
Proximal tMCAO (120 min, filament)	SD	i.p. injection of 50 or 500 ng rIL-6	rIL-6 reduced infarct volumes	IL-6R	[53]
Proximal pMCAO (electrocoagulation)	SD	i.c.v. injection of 2x50 or 2x500 ng rhIL-6 30 min prior to and again 15 min after occlusion	rhIL-6 reduced infarct volumes	IL-6R	[100]
IL-10 system					
Mouse					
Distal pMCAO (electrocoagulation)	C57BL/6	i.c.v. injection of 100 ng rmIL-10 5 min after occlusion	rmIL-10 reduced infarct volumes	IL-10R	[96]
Proximal tMCAO (60 min, filament)	C57BL/6	i.v. infection of IL-10-producing B cells 24 h prior to or 4 h after occlusion	IL-10-producing B cells reduced infarct volumes and reduced post-stroke inflammation	IL-10R	[16]
Rats					
Distal tMCAO (90 min, filament)	SD	i.v. injection of IL-10-overproducing mesenchymal stem cells 0 or 3 h after reperfusion	IL-10-overproducing mesenchymal stem cells reduced infarct volumes, improved motor functions and reduced inflammation	IL-10R	[119]
Distal pMCAO (photothrombotic)	SHR	i.c.v. injection of adenoviral vectors encoding human IL-10 (AdlIL-10) 90 min after occlusion	AdlL10 reduced infarct volumes and leukocyte infiltration	IL-10R	[130]
Distal pMCAO (electrocoagulation)	SHR	i.c.v. injection of 1 μg IL-10 30 min and 3 hours after occlusion and i.v. injection of 5 or 15 μg/h for 3 h starting 30 min after occlusion	IL-10 treatments reduced infarct volumes	IL-10R	[160]

*Ab* antibody, *BI* Barthel index, *bp* binding protein, *cTfRMAb* transferrin receptor monoclonal antibody, *GDNF* glial-derived neurotropic factor, *h* human, *i.c.v* intracerebroventricular, *IL* interleukin, *IL-1Ra* interleukin-1 receptor antagonist, *IL-1RI* interleukin-1 receptor 1, *IL-6R* interleukin-6 receptor, *IL-10R* interleukin-10 receptor, *i.p.* intraperitoneal, *i.v.* intravenous, *mAb* monoclonal antibody, *mRS* modified rankin score, *NIHSS* National Institutes of Health Stroke Scale, *pMCAO* permanent middle cerebral artery occlusion, *rh* recombinant human, *rm* recombinant mouse, *s.c.* subcutaneous, *SD* Sprague–Dawley, *SHR* spontaneously hypertensive rats, *solTNF* soluble tumor necrosis factor, *SVZ* subventricular zone, *tMCAO* transient middle cerebral artery occlusion, *tmTNF* transmembrane tumor necrosis factor, *TNF* tumor necrosis factor, *TNFR* tumor necrosis factor receptor

**Table 2:** Mechanistic profile of cytokine and cytokine receptor agonists/antagonists for use in experimental stroke

Drug name	Class	Structure	Specificity	References
Etanercept^a^ and biosimilars	Dimeric Fc-fusion protein	Hu TNFR2_exc_:IgG1-Fcγ1	solTNF, tmTNF, LTα3, & LTα2β1	
Infliximab^a^ and biosimilars	Monoclonal antibody	Mo/Hu chimeric IgG1/κ	solTNF & tmTNF	
Adalimumab^a^ and biosimilars	Monoclonal antibody	Hu IgG1/κ	solTNF & tmTNF	
Certolizumab pegol^a^	Monoclonal antibody fragment	PEGylated hu IgG1/κ Fab´	solTNF & tmTNF	
Golimumab^a^	Monoclonal antibody	Hu IgG1/κ	solTNF & (tmTNF)	
XPro1595	Dominant-negative inhibitor	TNF mutein	solTNF	[162]
XEN345	Dominant-negative inhibitor	TNF mutein	solTNF	[162]
cTfRMAb-TNFR	Fusion cTfR-protein	TNFR2_exc_:IgG1-cTfR	solTNF & tmTNF	[197]
R1antTNF	Inhibitor	TNFR1 selective mutein	TNFR1, solTNF?	[155]
DMS5540	Monovalent domain antibody	TNFR1-dAb:Albu-dAb	TNFR1	[108]
TROS	Dimeric nanobody	Hu TNFR1-Nb:Alb-70-96-Nb IgG1	TNFR1	[163]
ATROSAB	Monoclonal antibody	Hu IgG1	TNFR1	[88]
EHD2-scTNF_R2_	Dimeric single-chain fusion protein	Hu TNFR2:EHD2 IgE	TNFR2	[44]
TNCscTNF80	Trimerized single-chain fusion protein	Chicken TNC:huTNFR2	TNFR2	[25]
Anakinra^a^	Recombinant protein	IL-1Ra mutein	IL-1R1	
Rilonacept^a^	Dimeric fusion protein	Hu IL-1R1_exc_IL-1RAcP_exc_:IgG1-Fc	IL-1α & IL-1β	
Canakinumab^a^	Monoclonal antibody	Hu IgG1/κ	IL-1β	
MEDI-8968	Monoclonal antibody	Hu IgG2	IL-1R1	[21]
Gevokizumab	Monoclonal antibody	Hu IgG2/κ	IL-1β	[144]
LY2189102	Monoclonal antibody	Hu IgG4	IL-1β	[156]
XOMA 052	Monoclonal antibody	Hu IgG2/κ	IL-1β	[144]
IL-1RA-PEP	Fusion protein	IL-1Ra:PEP-1	IL-1R1	[195]
Tocilizumab^a^	Monoclonal antibody	Hu IgG1/κ	tmIL-6R & solIL-6R	
Siltuximab^a^	Monoclonal antibody	Mo/Hu chimeric IgG1/κ	IL-6	
Sarilumab^a^	Monoclonal antibody	Hu IgG1/κ	IL-6R	
Olokizumab	Monoclonal antibody	Hu IgG1/κ	IL-6, gp130	[154]
Elsilimomab	Monoclonal antibody	Hu IgG1/κ	IL-6	[184]
Sirukumab	Monoclonal antibody	Hu IgG1/κ	solIL-6	[190]
Clazakizumab	Monoclonal antibody	Hu IgG1/κ	IL-6	[110]
sgp130Fc (Olamkicept)	Fusion protein	Hu gp130_exc_:IgG1-Fc	IL-6/solIL-6R complex	[86]
Pegliodecakin (AM0010)	Pegylated recombinant protein	PEG-rHuIL-10	IL-10R	[118]
PEGylated-IL10	Pegylated recombinant protein	PEG-rMuIL-10	IL-10R	[50]

*Albu* anti-serum albumin, *cTfR* transferrin receptor, *dAb* domain antibody, *gp130* glycoprotein 130, *Hu* human, *IL* Interleukin, *IL-1R* interleukin-1 receptor, *IL-1Ra* interleukin-1 receptor antagonist, *IL-1RAcP* IL-1 receptor accessory protein, *LT*α lymphotoxin-alpha, *Mo* mouse, *solIL-6R* soluble interleukin-6 receptor, *solTNF* soluble tumor necrosis factor, *tmIL-6R* transmembrane interleukin-6 receptor, *tmTNF* transmembrane tumor necrosis factor, *TNC* tenascin, *TNF* tumor necrosis factor, *TNFR* tumor necrosis factor receptor

^a^FDA approved drug

### Tumor necrosis factor

The most extensively studied cytokine in experimental stroke is the proinflammatory and immune regulatory cytokine TNF. It exists in a secreted form (solTNF) and a transmembrane form (tmTNF), which is also involved in reverse signaling [87]. solTNF is derived from tmTNF, which is cleaved by the protease ADAM-17, also known as TNF-alpha converting enzyme (TACE) [14]. tmTNF and solTNF signals are transmitted through two distinct receptors, TNFR1 and TNFR2, that differ significantly both in cellular expression and downstream effects. Although solTNF binds both receptors with high affinity, it preferentially binds to TNFR1 (dissociation constant [*K*_d_] 20 pM) versus TNFR2 ([*K*_d_] ~ 400 pM), owing to a 30-fold faster dissociation rate from TNFR2 than from TNFR1 [69]. This has given rise to a ligand-passing hypothesis, stating that solTNF binding to TNFR2 is quickly passed to TNFR1. Binding by TNFRs to tmTNF or even TNF antagonists can induce reverse signaling through tmTNF, leading to cell activation, cytokine suppression, or apoptosis of the tmTNF-bearing cell (reviewed in [49]). While TNFR1 is expressed on virtually all cells, TNFR2 expression is restricted to cells of the immune system, glial cells, and endothelial cells. TNF’s proinflammatory effects are likely mediated through solTNF–TNFR1 signaling, leading to activation of two major, well-understood pathways. One leads to the induction of anti-apoptotic genes, mainly through activation of the transcription factor nuclear factor-kappa B (NF-κB). The second signaling pathway results in activation of cellular suicide programs, including the prototype of programmed cell death, apoptosis, but also the execution of programmed necrosis (necroptosis) [179]. Under physiological conditions, TNF does not induce cell death unless transcription, translation, or specifically the NF-κB pathway are blocked. Unlike TNFR1, TNFR2 is not associated with induction of apoptosis but preferentially promotes cell growth, and regeneration through NF-κB activation. TNFR1 can be activated by binding of either solTNF or tmTNF, whereas TNFR2 is only fully activated by tmTNF [68, 69]. A further level of complexity is added by the proteolytic cleavage of the extracellular domains of both TNFR1 and TNFR2 [182], which is increased upon TNFR activation (reviewed by [1]). The soluble TNFR1 and solTNFR2 ectodomains that are shedded can bind to TNF, albeit with low affinity, and can thus act as natural inhibitors of TNF.

Lymphotoxin-alpha (LTα), another TNFR agonist with important roles in immune regulation, also binds TNFR1 and TNFR2 and mainly mediates NF-κB-mediated signaling [134].

### Tumor necrosis factor in experimental stroke

The low baseline levels of TNF in the CNS under physiological conditions play an important role in neuronal function, by modulating glutamatergic synaptic transmission and plasticity [164]. Furthermore, TNF regulates neuronal networks involved in cognition and behavior [9], thereby attributing importance to TNF both in the healthy and stroke-lesioned CNS. Multiple checks are in place to finetune TNF’s production and activity, including regulation of *TNF* gene expression at transcriptional and translational levels, and the regulated shedding of TNF [117] and its receptors [135].

**Fig. 2:**
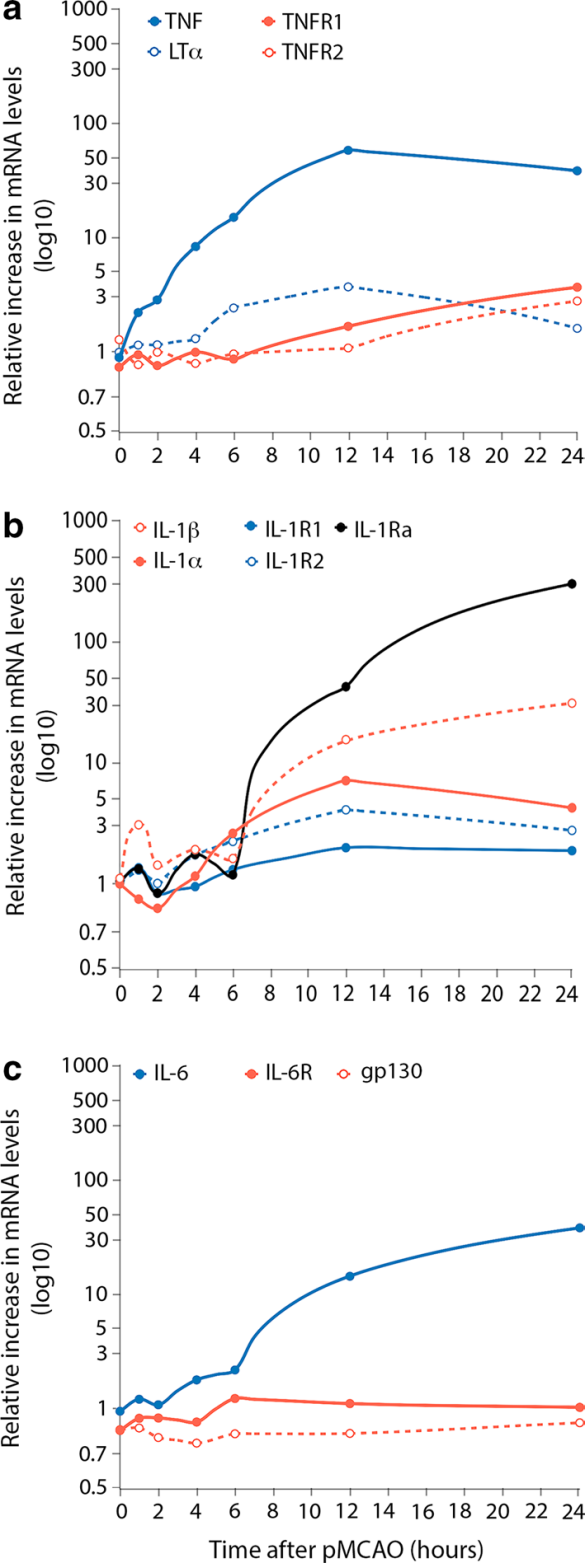
Temporal profile of cytokine and cytokine receptor upregulation in the acute phase after pMCAO. **a** Graphical presentation of the temporal profile of TNF, LTα, TNFR1, and TNFR2 mRNAs in the same ischemic hemispheres from mice subjected to pMCAO. **b** Graphical presentation of the temporal profile of IL-1β, IL-1α, IL-1Ra, IL-1R1, and IL-1R2 mRNAs after pMCAO. **c** Graphical presentation of the temporal profile of IL-6, IL-6R, and gp130 mRNAs after pMCAO. Data are presented as relative increases in mRNA levels compared with unmanipulated controls. TNF, TNFR1 and TNFR2 mRNA data have been obtained from [93, 94], whereas LTα mRNA data are unpublished data performed on the same experimental mice and conditions as [94]. The sequence of the LTα *TaqMan* probe was AGGAGGGAGTTGTTGCTCAAAGAGAAGCCA, for the LTα sense primer it was CTGCTGCTCACCTTGTTGGG, and for the LTα antisense primer it was TAGAGGCCACTGGTGGGGAT. IL-1α, IL-1β, IL-1Ra, IL-1R1, and IL-1R2 mRNA data have been obtained from [33]. IL-6, IL-6R, and gp130 mRNA data have been obtained from [70]. Note the logarithmic *Y* axis. *gp130* glycoprotein 130, *IL* interleukin, *IL-6R* interleukin-6 receptor, *LT*α lymphotoxin-alpha, *TNF* tumor necrosis factor, *TNFR* tumor necrosis factor receptor

A particular role of TNF/TNFR1 in the etiopathogenesis of stroke is suggested by genome-wide association studies that found a polymorphism in the *TNF* gene that increases the susceptibility for stroke [178]. After pMCAO, TNF is acutely and significantly upregulated, peaks at 12–24 h (Fig. 2a), and remains elevated for days (Fig. 1d), making TNF a key player both in acute and chronic ischemia and in post-ischemic neuronal plasticity (reviewed by [91]). TNF is primarily produced by microglia in the early phase after experimental stroke and sustained by macrophages at later time points [20, 32, 92, 94], although other cell types like ependymal, astroglial and neuronal cells have also been reported to produce TNF during ischemic conditions (reviewed by [91]).

The use of genetically modified mice has been invaluable for establishing the role of TNF in the pathogenesis of ischemic stroke. Conventional TNF-knock out (KO) mice [92] and conditional TNF-KO mice with ablation of TNF in myeloid cells, including microglia [31] have larger infarcts and worse behavioral deficits than control mice after pMCAO. This suggests a neuroprotective role of microglial-derived TNF in ischemic stroke, an effect which appears to be mediated via TNFR1 [92, 170]. Interestingly, mice with a loss of TACE-mediated cleavage preventing shedding of solTNF (and thus expressing only tmTNF) develop smaller infarcts than their littermates [104], suggesting that removal of solTNF but preservation of tmTNF is neuroprotective in ischemic stroke.

Finally, a polymorphism in the LTα gene (*LTA*) has been linked to increased susceptibility for stroke [178], suggesting that also LTα plays a role in the etiopathogenesis of stroke. However, LTα levels appear to remain relatively constant in the acute phase after pMCAO in mice (Fig. 2a, Lambertsen et al., unpublished data), suggesting that brain-derived LTα has no major role in the inflammatory response post-stroke.

### Anti-tumor necrosis factor treatment in experimental stroke

The currently used FDA- and EMA-approved anti-TNF therapeutics block both solTNF and tmTNF (Table 2). These therapeutics appear to relieve fatigue and symptoms of depression that can be associated with chronic inflammatory diseases [177]. Despite reports of improved neurological outcome in patients with stroke or traumatic brain injury who are treated with perispinal etanercept [172, 174] (Table 1), none of the currently used anti-TNF therapeutics have so far been approved as a neuroprotective strategy in combination with tissue plasminogen activator treatment. This may be because targeting both solTNF and tmTNF can predispose patients to both cardiovascular and demyelinating diseases [151], which is in line with the finding that a single nucleotide polymorphism in the TNFR1 gene (*TNFRSF1A)* that mimics the effect of anti-TNF therapeutics, is a risk factor for developing multiple sclerosis [67]. In combination with the observation that not only TNF-KO mice but also TNF-R1 KO mice develop larger infarcts than wild-type mice [92, 170], this calls for precaution in using the currently approved anti-TNF therapeutics and emphasizes the need for more specific anti-TNF therapeutics.

**Fig. 3:**
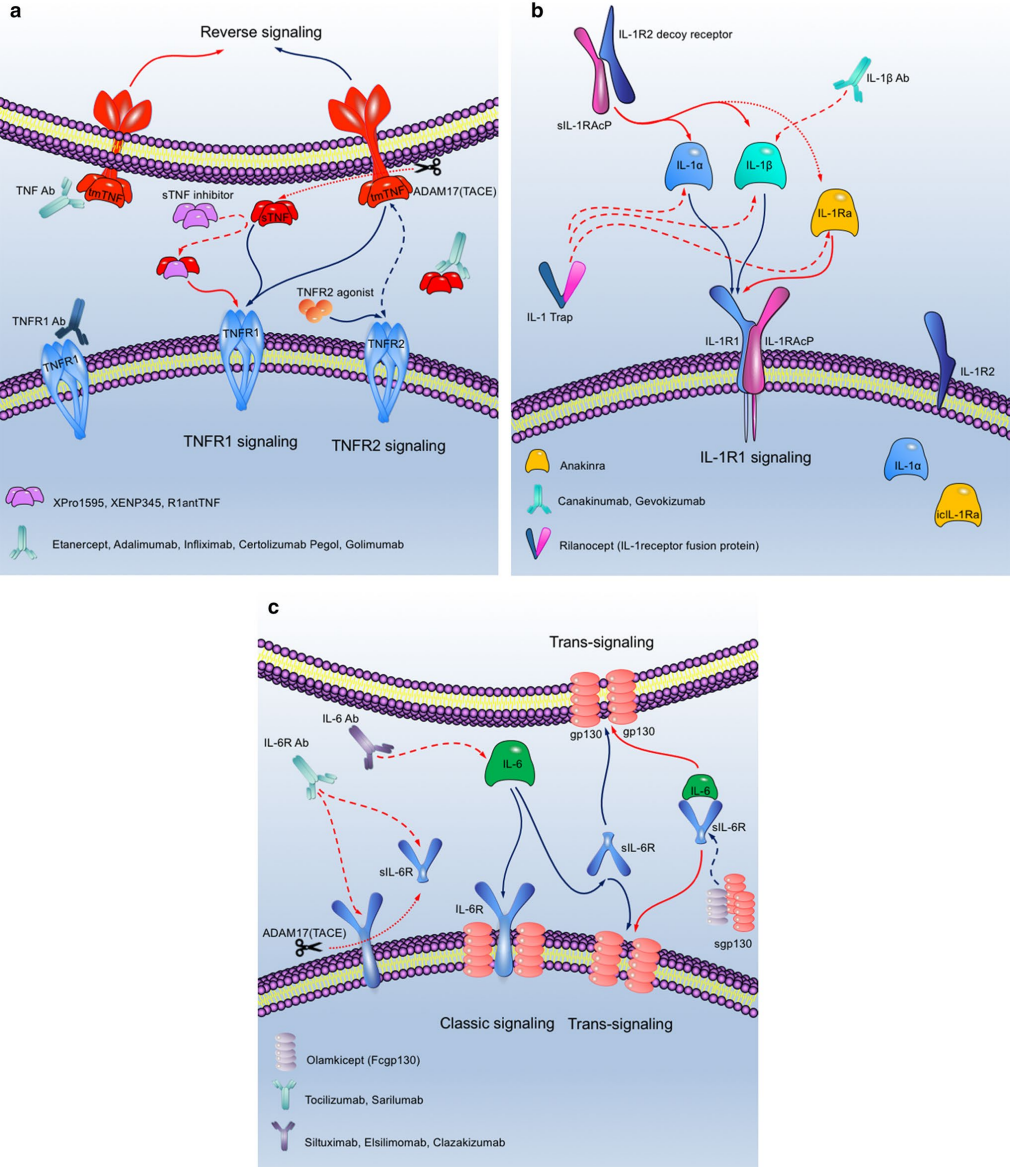
Schematics presenting mechanisms of actions of approved and selected experimental cytokine and cytokine receptor agonists and antagonists. **a**–**c** TNF (**a**), IL-1 (**b**), and IL-6 (**c**) signaling via their receptors and mechanisms of actions of approved and selected novel inhibitors. Figures are modified using Protein Lounge Pathway Database (www.proteinlounge.com). *Ab* antibody, *gp130* glycoprotein 130, *icIL-1Ra* intracellular interleukin-1 receptor antagonist, *IL* interleukin, *IL-1Ra* interleukin-1 receptor antagonist, *IL-1R1* interleukin-1 receptor type 1, *IL-1R2* interleukin-1 receptor type 2, *IL-1RAcP* IL-1 receptor accessory protein, *sIL-1RAcP* soluble IL-1 receptor accessory protein, *IL-6R* interleukin-6 receptor, *sgp130* soluble glycoprotein 130, *solIL-6R* soluble interleukin-6 receptor, *solTNF* soluble tumor necrosis factor, *tmTNF* transmembrane tumor necrosis factor, *TNF* tumor necrosis factor, *TNFR* tumor necrosis factor receptor

There has been little preclinical testing of therapeutics that exclusively target solTNF (XPro1595, XEN345, and possibly R1antTNF) (Tables 1, 2 and Fig. 3a) and leave signaling via tmTNF–TNFR1/2 intact. A comparative study of a single i.v. dose of XPro1595 (a dominant-negative solTNF inhibitor) or etanercept, administered at a dose of 10 mg/kg, 30 min after pMCAO, showed that both compounds affected the inflammatory response and improved motor functions and motor learning skills compared to vehicle 1 and 5 days after pMCAO, but had no effect on infarct volume [30]. This indicates that targeting solTNF alone may be efficient for the treatment of post-stroke inflammation. Similarly, recent findings showed that topical, but not systemic administration, of XPro1595 can rescue tissue at risk after experimental spinal cord injury, while etanercept had no effect [129], suggesting that topical administration of XPro1595 can inhibit solTNF present locally in the CNS. Clearly, more studies are needed to clarify whether XPro1595 is able to rescue tissue at risk in the peri-infarct. However, given the prevalence of post-stroke infections in humans, leaving tmTNF signaling intact may decrease the risk of infections.

While it seems relevant to retain the neuroprotective TNFR1 signaling in the acute phase after stroke, TNFR1 also plays a role in sustaining chronic inflammation in mouse models of multiple sclerosis and TNFR2 is important for remyelination [18]. Although more studies are clearly required to clarify the role of neuronal TNFR1 signaling in the acute phase post-stroke, it is possible that TNFR1-specific antagonists [R1antTNF, DMS5540, TROS (*TNF receptor one silencer*), ATROSAB (*antagonistic TNF receptor one*-*specific antibody*)] (Table 2) that preserve TNFR2 signaling, will be important in improving neuronal and synaptic remodeling in the chronic phase of stroke.

Due to their large size, many biologic TNF inhibitors do not cross the BBB and must be modified to enable BBB penetration and access to the brain parenchyma. One such drug is cTfRMAb-TNFR (Table 2), which ferries TNFR across the BBB using the transferrin receptor (TfR) [197]. In a preclinical study, i.v. injection of cTfRMAb-TNFR was compared to etanercept in a tMCAO model and when administered 90 min after occlusion resulted in reduced infarct volumes and reduced neural deficit 1 and 7 days post-stroke, whereas etanercept had no effect [167](Table 1). Despite the fact that both cTfRMAb-TNFR and etanercept are TNFR2 fusion proteins, the authors ascribed the beneficial effect of cTfRMAb-TNFR to the modification of this protein to allow it to be transported across the BBB [15].

In another preclinical study, sTNF-α R1 (solTNFR1) (Table 2) administered by intracortical infusion for 1 week after photothrombotic stroke was found to preserve deprivation-induced axonal plasticity in the cerebral cortex post-stroke [98] (Table 1). This effect was ascribed to sTNF-α R1 competing for solTNF with TNFR1 receptors, supporting the hypothesis that ablating solTNF is beneficial in ischemic stroke. This is in line with a preclinical study showing that intra-arterial injection of solTNFR1-overexpressing dendritic cells 6 h after tMCAO reduces infarct size and inflammation 3 days post-stroke [186] (Table 1).

### Interleukin-1

The IL-1 family comprises 11 members (IL-1α, IL-1β, IL-1 receptor antagonist (IL-1Ra), IL-18, IL-33, IL-36α, IL-36β, IL-36γ, IL36-Ra, IL-37, and IL-38), forming a network of proinflammatory cytokines that regulate innate immune cells and function as key players in inflammation (review by [43]). Despite structural and functional similarities and evidence of a common ancestry [143], so far only IL-1α, IL-1β, and IL-1Ra have been studied extensively in ischemic stroke.

Both IL-1α and IL-1β are expressed and translated as precursor (pro) proteins. ProIL-1α is biologically active, but it lacks the signal peptide that allows it to leave the cell [143]. IL-1α is a ‘dual-function’ cytokine with both nuclear and cytoplasmic functions, but danger signals from necrotic cells can promote the secretion of IL-1α [48], causing neutrophil recruitment and exacerbation of inflammation [24]. Apoptosis causes IL-1α to translocate to the nucleus, where it binds to chromatin, a mechanism which is known to restrain inflammation [34]. IL-1α is considered to be an early danger signal that modulates a wide range of inflammatory reactions through the interleukin-1 receptor type 1 (IL-1R1) [48, 143]. Following injury, the proteolytic cleavage of IL-1α occurs through the actions of calpain, and possibly inflammasomes [194]. Membrane-bound, unprocessed IL-1α acts in a paracrine fashion on IL-1R expressing cells [42] to modulate angiogenesis, cell proliferation, senescence, apoptosis, and migration, and cytokine production ([149] and review by [43]).

In contrast to proIL-1α, proIL-1β is a biologically inactive protein, and both proIL-1β and mature IL-1β appear extracellularly [143], indicating that processing can take place after secretion. ProIL-1β is cleaved by caspase-1 (or IL-1 converting enzyme) [143], which gets activated by the assembly of the inflammasome, a process triggered in turn by damage-associated molecular pattern signals [72]. ProIL-1β can also be cleaved by neutrophil serine proteases such as proteinase 3 and elastase [123].

The natural regulator of IL-1 is IL-1Ra, which is found in two structural variants, secreted (s)IL-1Ra and intracellular IL-1Ra (icIL-1Ra), that both target the IL-1R1 [6]. The icIL-1Ra isoform is less explored but believed to exert multiple functions inside the cell [6], such as modulating the effect of IL-1α and/or acting as regulator of proIL-1β [102]. IL-1Ra is expressed by monocytes/macrophages, neutrophils [105], microglia [33], and other cells [42].

IL-1α/β induce their biological effects through IL-1R1, which is expressed in low numbers (< 100) on nearly all cells in the brain [42]. Binding of IL-1 to IL-1R1 allows the binding of the interleukin-1 receptor accessory protein (IL-1RAcP, IL-1R3), which is a key component of the receptor/agonist signaling complex [6, 143]. Recruitment and binding of IL-1RAcP converts the low-affinity binding between IL-1R1 and IL-1 to a high-affinity binding allowing further signal transduction [65]. IL-1 signaling is complex but potent with < 10 receptors/cell required to be occupied before a full response is triggered [166]. This means that IL-1Ra needs to be present in 100–1,000-fold molar excess to control its biological properties [42].

IL-1R2 shares structural characteristics with IL-1R1, but it lacks the cytoplasmic domain that allows signal transduction. IL-1R2 binds IL-1 as a decoy receptor [42, 143]. IL-1R2 is expressed by the same cells as IL-1R1 but is particularly abundant on monocytes, and neutrophils [42, 45]. IL-1R2 binds IL-1α in the cytosol, preventing its interaction with IL-1R1 when released from necrotic cells [196]. All the IL-1Rs are also found in a soluble form [90].

### Interleukin-1 in experimental stroke

IL-1 is a major player in stroke pathology (Fig. 1g, h). As for the *TNF* gene, a polymorphism in the *IL*-*1A* gene has been associated with an increased susceptibility for ischemic stroke [199] whereas a polymorphism in the *IL*-*1B* gene has been associated with lower stroke risk [13], although this is still controversial [193, 199]. Polymorphisms in the *IL1RN* gene do not affect the risk for stroke [199], but increased plasma IL-1α combined with a polymorphism in the *IL1RN* gene increases the risk of post-stroke infection [10].

So far, focus has been on understanding the role of IL-1β in experimental stroke models, however data suggests that also IL-1α, which is significantly upregulated in mice 6–24 h after pMCAO (Fig. 2b) [33] and 7 days after tMCAO [149], plays an important role in stroke-induced neuroinflammation [33, 171]. Following experimental stroke in rodents, IL-1α was shown to be expressed by platelets and microglia [33, 40]. The presence of platelet-derived IL-1α acutely (6 h) after experimental stroke [33] supports findings that IL-1α drives neurovascular inflammation and facilitates neutrophil infiltration into the ischemic brain [171]. At 24 h after pMCAO, microglia are the key producers of IL-1, with approximately 50% of the IL-1α producing microglia co-expressing IL-1Ra and 17% co-expressing IL-1β, demonstrating that IL-1β and IL-1α are largely produced by segregated populations of microglia in the ischemic brain [33]. It is, therefore, likely that IL-1α in platelets in addition to few IL-1α/β producing microglia impacts the balance between IL-1/IL-1Ra early after stroke onset [33]. Findings that IL-1α and IL-1Ra are co-expressed in microglia support the view that icIL-1Ra can regulate the action of intracellular IL-1α [113].

IL-1β is constitutively expressed in the CNS [42] where it exerts neurotrophic factor-like activity [161] or regulates both the expression and activity of ion channels [181]. IL-1β is upregulated acutely after ischemic stroke (Fig. 1)[32, 33, 37] and peaks at 12-24 h (Fig. 2b) primarily in microglia and macrophages [32, 37], and later in astroglial-like cells [183].

IL-1 has been shown to aggravate stroke pathology (Table 1) as demonstrated by findings in transgenic mice overexpressing a dominant-negative form of caspase-1 in neurons [54], caspase-1 KO mice [73], and IL-1α/β KO mice [17], which all show reduced infarct volumes after experimental stroke. Additional support comes from early studies demonstrating that administration of recombinant IL-1β exacerbated damage [99] as does intracerebroventricular (i.c.v.) delivery of an IL-1Ra antiserum [101]. Systemic administration of IL-1β just before tMCAO worsened outcome in rodents through neutrophil- and platelet-dependent mechanisms reducing reperfusion [109].

In addition, IL-1Ra is an acute phase protein [55] that blocks the action of IL-1. Administration of IL-1Ra reduced ischemic brain damage after both tMCAO and pMCAO in rats [59, 137] and mice [175] (Table 1) and IL-1Ra-overexpressing mice show reduced infarct volumes, whereas IL-1Ra KO mice display increased infarct volumes compared to littermate mice after pMCAO [33].

### Anti-interleukin-1 treatment in experimental and human ischemic stroke

IL-1Ra is the only therapeutic agent directed against IL-1-induced inflammation (Fig. 3b) that has been tested in randomized clinical trials in ischemic stroke (Table 1). In pre-clinical stroke models, recombinant (r)IL-1Ra is protective after central [137] and peripheral [59] administration and, similar to i.c.v. injection of anti-IL-1β antibody (Ab) [191] or IL-1Ra, was shown to reduce infarct volumes after MCAO in rats [99, 137] and pMCAO in mice [121].

Although IL-1Ra can reach the brain after systemic administration in the rat [66] and modulates long-term functional recovery after experimental stroke [62], its use in stroke patients has proven challenging. Pharmacokinetic studies have shown that rIL-1Ra crosses the BBB slowly [71] and has a very short half-life in the circulation [64], and thus it is difficult to achieve therapeutic IL-1Ra concentrations in the brain [57].

The first randomized, double-blind, placebo-controlled trial using i.v. injected recombinant human (rh)IL-1Ra in acute stroke patients (given within the first 6 h of stroke onset) showed a reduction in neutrophil count, plasma CRP, and IL-6 compared to placebo, and exploratory efficacy analysis indicated that patients receiving rhIL-1Ra had minimal to no disability three months after stroke [51]. Recently, the SCIL-STROKE (subcutaneous interleukin-1 receptor antagonist in ischemic stroke) phase II trial, using subcutaneous (s.c.) injections of IL-1Ra in combination with i.v. thrombolysis, showed reduced plasma IL-6 levels, whereas neurological recovery three months after stroke was unaffected [159]. Exploratory efficacy analysis suggested that the expected beneficial effect of IL-1Ra on clinical outcome by reducing inflammation might have been counteracted by a negative effect, which could represent an interaction with alteplase [159].

### Interleukin-6

Another potent proinflammatory cytokine with pleiotropic functions is IL-6, which is expressed on many cell types, including monocytes, neurons and glial cells (Fig. 1j, k)[52, 70]. The pleiotropism of IL-6 may be explained by IL-6 eliciting fundamentally different cellular responses depending on whether the classic or the trans-signaling pathway is activated [152]. This depends on the IL-6 receptor system that consists of the IL-6 receptor (IL-6R) as well as soluble IL-6R (sIL-6R) and glycoprotein 130 (gp130), which due to its cytoplasmic domain is responsible for the signal transduction. Soluble IL-6R is formed by cleavage from the IL-6R by TACE/ADAM17 [141] or by translation of different IL-6R mRNA splice variants [103].

In classic signaling, IL-6 binds to and forms a complex with membrane-bound IL-6R, which then recruits gp130. Trans-signaling occurs when IL-6 binds sIL-6R, which then binds to membrane-anchored gp130 [141]. Unlike IL-6R, which is expressed by neurons, microglia, neutrophils, monocytes, hepatocytes and CD4^+^ T cells and thus limits classic signaling to only a few tissues [58], gp130 is ubiquitously expressed in the body (reviewed by [145]), increasing the spectrum of IL-6 target cells. Trans-signaling is normally tightly regulated [185] and can be counteracted by a soluble form of gp130 (sgp130), which is generated by alternative splicing of gp130 mRNA and is present in serum [85]. Once IL-6 is released into the blood it can bind sIL-6R but also sgp130 [150], which immediately interferes with IL-6 trans-signaling [58]. As sgp130 levels are much higher than sIL-6R, trans-signaling does not occur under physiological conditions.

Classic IL-6 signaling is believed to be anti-inflammatory and protective [185], while trans-signaling is responsible for the pro-inflammatory effects mediated by IL-6 [147, 152].

### Interleukin-6 in experimental stroke

IL-6 is expressed in the normal CNS, where it influences neuronal homeostasis by acting as a neurotrophic factor via the classical signaling pathway (reviewed by [147]). Ischemic stroke in mice and rats leads to a significant increase in the levels of IL-6 from 6 to 12 h (Fig. 1 and 2c), and in both IL-6R and gp130 from 3 days [3, 70]. IL-6 has been shown to be neuroprotective in experimental stroke [192] although this is still debated [29]. In human stroke, IL-6 serum levels increase within the first 24 h and have been shown to correlate significantly with infarct size and survival [11, 157]. A similar correlation has not been observed for sIL-6R [46, 70]. While studies of IL-6 expression in the ischemic brain post-mortem are sparse, one study showed that IL-6 levels were elevated in the infarct already in the acute phase after stroke and remained elevated at later time points [126]. Supporting the neuroprotective effect of brain-derived IL-6 are findings showing a positive effect of IL-6 on post-stroke neurogenesis, leading to long-term functional recovery [111].

### Anti-interleukin-6 treatment in ischemic stroke

Similar to patients treated with nonspecific TNF antagonists, non-neurological patients treated with IL-6 inhibitors are at increased risk of infections (reviewed in [169]). Clinical stroke studies show that sIL-6R correlates with the degree of leukocyte infiltration [85] and that sIL-6R neutralizing antibodies are beneficial [146]. In comparison, anti-IL-6R antibodies target both the membrane-bound form of IL-6R and sIL-6R, and therefore, affect classical and trans-signaling equally (Fig. 3c and Table 2).

If classical IL-6 signaling is protective and trans-signaling detrimental, selective neutralization of the potential, detrimental trans-signaling is possible by administration of the chimeric protein sgp130Fc (Fig. 3c and Table 2). Sgp130Fc is a fusion protein that contains the extracellular domain of human gp130 and the Fc-fragment of human IgG1. This allows sgp130Fc to bind to the IL-6/solIL-6R complex, but not to sIL-6R alone [86], whereby spg130Fc blocks trans-signaling [52] (Fig. 2c). Such specific inhibition of the trans-signaling pathway using, i.e. sgp130, which does not compromise classic signaling, could be a promising therapeutic tool in future stroke research.

### Interleukin-10 in clinical and experimental stroke

IL-10 is a pleiotropic anti-inflammatory cytokine mainly produced by type-2 helper T cells, which in turn regulate inflammatory reactions. IL-10 binds to IL-10 receptors (IL-10R) to reduce inflammation and limiting apoptosis [148]. In the CNS, astrocytes, neurons, and microglia have been reported to produce IL-10 [114, 188].

A meta-analysis investigating the association of *IL10* gene polymorphism with the risk of ischemic stroke showed no overall significant association between IL-10 and the risk of ischemic stroke, but an association was found with large vessel disease and small vessel disease [89], suggesting that some subtypes of ischemic stroke are associated with *IL10* gene polymorphisms.

In experimental stroke, IL-10 mRNA and protein and IL-10R mRNA levels are increased, with IL-10 noted in microglia and IL-10R on astrocytes in the peri-infarct area [126, 132]. In transgenic mice overexpressing IL-10, infarct volumes were reduced, and apoptosis decreased 4 days after pMCAO [38]. Furthermore, low IL-10 levels were associated with poor stroke outcome and a delayed, exacerbated inflammatory response after pMCAO that was ameliorated by IL-10 administration after pMCAO [132] (Table 1). Therapeutic administration of IL-10 has been shown to be neuroprotective in experimental stroke and to limit post-stroke inflammation [96, 97, 130, 139, 160, 165] (Table 1),

Low plasma IL-10 levels in patients with subcortical or lacunar stroke are associated with neurological worsening within the first 48 h [180], attributing IL-10 a role in the acute neuroinflammatory response after stroke. This is in line with findings by Protti et al. showing that patients with low IL-10 levels deteriorated neurologically within the first 3 days post-stroke [136]. Stroke patients are prone to infection due to stroke-induced immunodepression, however, and increased serum IL-10 levels have been identified as an independent predictor of post-stroke infection [22, 187]. Women have poorer recovery after ischemic stroke than men, even after controlling for age and stroke severity [19, 80]. This may be partly due to the increased IL-10 levels 24 h post-stroke and an associated higher incidence of post-stroke urinary tract infection and poorer overall outcomes in women have been suggested to be a contributing factor [35]. Overall, these studies indicate that an excessive IL-10 response can lead to post-stroke immunosuppression and worsen neurological outcome, suggesting that IL-10 therapeutics should be given with caution. Future studies should be aimed at differentiating between central and peripheral IL-10 effects post-stroke.

## Concluding remarks

The dual role of inflammation in both injury and repair complicates attempts to target inflammatory signals in stroke patients. “Single-target” therapies appear insufficient because ischemic stroke involves several mechanisms. Therapeutic approaches should, therefore, most likely target several cell types and different post-ischemic phases to promote protection and recovery.

A possible new approach is to enhance proinflammatory cytokine inhibition either by simultaneous targeting of more than one cytokine or using a more selective targeting approach where only part of the signaling cascade initiated by a given cytokine is inhibited. More selective targeting can be achieved because some of the detrimental and beneficial signals diverge at the level of ligand (e.g. solTNF or tmTNF and IL-1 or IL-1Ra) and at the level of the receptor (e.g. TNFR1 or TNFR2 and IL-6R or sIL-6R). Accordingly, specific inhibition of solTNF, IL-1, or IL-6 trans-signaling might be sufficient to inhibit the pathological consequences of deregulated cytokine signaling while leaving beneficial signaling pathways intact.

The differential roles of cytokine and cytokine receptors, and the function of cytokines derived from specific cell subsets make it clear that the use of anti-cytokine drugs can be improved or adjusted to the specific disease context. A novel approach to block detrimental inflammation following experimental ischemia is the use of cell-type-restricted targeting of cytokines, or the creation of Activity-on-Target cytokines (AcTakines), which is immunotherapy consisting of mutated cytokines with reduced binding affinity coupled to a targeting moiety that guides cytokines to the desired cell target [60]. Recently, Nedospasov and colleagues designed myeloid cell-specific TNF inhibitors (MYSTIs), which are recombinant mini-antibodies with dual specificity, that can bind to the surface molecule F4/80 or CD11b on myeloid cells and to solTNF and were found to be beneficial in in vivo models of acute hepatotoxicity and arthritis [47, 128].

For anti-inflammatory therapies to be successful in stroke treatment, a better understanding is needed of both the temporal and spatial dynamics of resident microglia and recruited inflammatory cells. Despite intense investigation, there are still numerous controversies concerning the time course of leukocyte recruitment in acute stroke. An improved understanding of the heterogeneity of the inflammatory response in this disease also demands better imaging studies of stroke patients, using tracers to identify both infiltrating cells and functional, relevant cytokine receptors. The heterogenic roles that microglia play in stroke make it challenging to identify strategies that modulate microglial function, but promising results of pre-clinical studies suggest that this should be a major focus of attention in future stroke research.

As evidenced above, post-stroke neuroinflammation is both a tool and a target for therapy. However, care must be taken as to when, where, and how to intervene with neuroinflammatory responses. Taken altogether, this calls for further translational stroke research.

## Abbreviations

Ab: Antibody
ATROSAB: Antagonistic TNF receptor one-specific antibody
BBB: Blood brain barrier
DWI: Diffusion-weighted imaging
gp130: Glycoprotein 130
icIL-1Ra: Intracellular interleukin-1 receptor antagonist
IL: Interleukin
IL-1R: Interleukin-1 receptor
IL-1Ra: Interleukin-1 receptor antagonist
IL-1RAcP: Interleukin-1 receptor accessory protein
IL-6R: Interleukin-6 receptor
IL-10R: Interleukin-10 receptor
i.c.v.: Intracerebroventricular
i.v.: Intravenous
ko: Knock out
LTα: Lymphotoxin-alpha
MCA: Middle cerebral artery
MCAO: Middle cerebral artery occlusion
NF-κB: Nuclear factor-kappa B
PET: Positron emission tomography
pMCAO: Permanent middle cerebral artery occlusion
PMN: Polymorphonuclear
PWI: Perfusion-weighted imaging
r: Recombinant
rh: Recombinant human
s.c.: Subcutaneous
sgp130: Soluble glycoprotein 130
sIL-1Ra: Secreted interleukin-1 receptor antagonist
sIL-6R: Soluble interleukin-6 receptor
solTNF: Soluble tumor necrosis factor
TACE: Tumor necrosis factor-alpha converting enzyme
TfR: Transferrin receptor
tmTNF: Transmembrane tumor necrosis factor
TNF: Tumor necrosis factor
TNFR: Tumor necrosis factor receptor
tMCAO: Transient middle cerebral artery occlusion
TROS: TNF receptor one silencer
